# Women’s experiences of pregnancy after gastric bypass surgery

**DOI:** 10.18332/ejm/151550

**Published:** 2022-08-04

**Authors:** Li Thies-Lagergren, Azin Mårtensson, Anahita Safi

**Affiliations:** 1 Department of Midwifery Research, Reproductive, Perinatal and Sexual Health, Department of Health Sciences, Faculty of Medicine, Lund University, Lund, Sweden

**Keywords:** pregnancy, midwives, qualitative study, gastric bypass surgery, maternal experience

## Abstract

**INTRODUCTION:**

Globally, 20% of women who become pregnant are obese at the time of conception. The prevalence of women becoming pregnant after gastric bypass (GBP) surgery has been increasing. Little is known regarding women’s experiences of pregnancy after GBP surgery and midwives can expect to care for an increasing number of women with prior GBP surgery. Midwives play an important role in supporting these women. The aim of this study was to describe women’s experiences of pregnancy after gastric bypass surgery.

**METHODS:**

This was a qualitative descriptive interview study using content analysis including 13 women who had a pre-pregnancy GBP surgery. Women were recruited at antenatal clinics, a specialist maternity care unit, and via social media.

**RESULTS:**

Three categories emerged: ‘Importance of support’, which described the nature of support from midwives; ‘The presence of the baby in the womb’, which described the mother’s relationship to the unborn baby; and ‘Aggravating circumstances’, which described physical circumstances challenging the pregnancy and the experience of it.

**CONCLUSIONS:**

Women who became pregnant after GBP surgery described ambivalent feelings about their pregnant selves. More knowledge is needed in how prior GBP surgery affects pregnant women emotionally. The study found that specific training and guidelines for the care by midwives are warranted for this group of women. Employers should ensure highly competent midwives to care for pregnant women with pre-pregnancy GBP surgery. To increase the knowledge on women’s childbearing experiences after a GBP surgery, more research with a qualitative design is needed, as there is currently a large research gap on the topic.

## INTRODUCTION

Twenty percent of women who become pregnant globally are obese at the time of conception^1,2^. Obesity is classified as having a Body Mass Index (BMI) ≥30 kg/m^2^ and is a chronic disease^3^. These women are seen as being at increased risk of suboptimal maternal and infant outcomes^4,5^. For women with morbid or severe obesity, gastric bypass (GBP) surgery is now widely used to reduce this risk. Research has focused on identifying the risks and benefits of employing GBP^6,7^. GBP surgery results in massive weight loss and can reduce the risk of developing gestational diabetes, hypertensive disorders or experiencing cesarean section, vacuum extraction, or postpartum hemorrhage. There is an ever-expanding literature on the medical risks and benefits of GBP for women who plan to become pregnant, with benefits including an increased perception of body control^8^ and substantial sustainable improvement in quality of life^7^. Physical complications such as small bowel obstruction, ileus and dumping syndrome with subsequent hypoglycemia are associated with GBP^7,9^. These complications can lead to reduced dietary intake and lower absorption of food, which subsequently may lead to malnutrition during pregnancy and should be considered as leading to a high-risk pregnancy^2^. For the infant an increased risk of being born small for gestational age (SGA), with congenital anomalies, preterm or needing to be transferred to a neonatal intensive care unit (NICU) has been reported^9^ and decreased initiation of breastfeeding has also been reported^10^.

A review of the literature on care for this group of women by midwives revealed a knowledge gap in relation to women’s experience of pregnancy and GBP. Where midwives have primary responsibility for care, as in Sweden, it is important for midwives to know about medical and emotional challenges that pregnant women who have undergone prior GBP surgery may face at every stage of pregnancy and birth, because midwives have a responsibility to make sure that all women attain a positive pregnancy experience, including physical and sociocultural normality, a healthy pregnancy for mother and baby, a transition to a positive labor and birth, and to achieve a positive motherhood^11^. Thus, the aim of this study was to describe women’s experiences of pregnancy after gastric bypass surgery.

## METHODS

This study is based on qualitative descriptive interviews that used an inductive approach and content analysis^12^.

### Participants and settings

The study is based on interviews of women who became pregnant after GBP surgery. To be eligible for the study, women had to be aged ≥18 years, understand Swedish, undergone GBP surgery before conception of the latest pregnancy, and given birth within the last 10 years prior to recruitment.

In Sweden, the midwife is the primary healthcare provider throughout the childbearing process. If complications arise, the midwife works together with an obstetrician, and a care plan is established that considers the pregnant woman’s health status and her medical and reproductive history. All women in Sweden who have undergone pre-pregnancy GBP surgery are followed-up by a multidisciplinary team (specialist physician, surgeon, nutritionist, psychiatrist, and other specialists as required). National guidelines regarding post-bariatric prenatal care are currently lacking in Sweden.

### Recruitment

Eligible women were recruited at antenatal clinics and specialist maternity-care centers. Potential participants were also approached at online forums and Facebook^©^ groups. Recruiting from different settings ensured that a variety of potentially eligible patients could be invited^13^. Potential subjects could e-mail the researchers to ask for information, and the researchers could then return detailed information about the study. The date and location of meeting between patient and researcher were set according to the prospective participant’s wishes.

Potential participants were given printed information about the study and were told that participation was entirely voluntary. All who were selected were told that they were free to withdraw without having to explain. The participants who were interviewed by telephone gave oral consent and received a consent form sent together with a pre-paid return envelope. Written consent was obtained from all participants.

Initially, a total of 15 women agreed to participate. Nine women were recruited via Facebook^©^ and two women from an online forum. One woman was recruited from an antenatal clinic, and finally three women were recruited from a specialist maternity care center. Two informants recruited via social media opted out of the study because they said they could not take time to participate. The final cohort consisted of four primiparous women and nine multiparous women.

### Data collection

Data collection was conducted in February and March 2018. According to participants preferences, eleven interviews were conducted via telephone so they would not have to visit an interview location and two interviews were conducted in face-to-face meetings at a specialist maternity care center. Two members of the research team (AS and AM) conducted all the interviews. The first author (LTL) examined and validated the first two interviews. Interviews lasted 25– 45 minutes, with an average of 30 minutes, were digitally recorded and transcribed verbatim. The interviews were coded so that only the authors could determine who the informants were.

Participants were asked to elaborate on and talk freely about their experience of pregnancy after GBP surgery, based on semi-structured interviews with one open question: ‘You had the gastric bypass surgery and then became pregnant, tell us about your experience of pregnancy’. Exploratory questions such as: ‘Could you say some more about that?’, ‘What do you mean by that?’ or ‘Will you give some examples?’ were used to encourage participants to tell more, if clarification were needed. Also, if not mentioned by the responders during the interviews, further questions to get a deeper understanding were posed such as: ‘How did you experience the contact with your midwife?’, ‘How did you experience the surroundings?’ or ‘How did you react to gaining weight during pregnancy?’.

### Data analysis

Initially, AS and AM read and re-read the interviews, separately, in the preparation phase in order to familiarize and immerse themselves in the data, as well as to acquire a general sense of the nature of the material^12^. Subsequently, the material was reviewed in order to judge the degree of meaningfulness in relation to the research question. Transcription and initial analyses of the text were completed by AS and AM, individually.

Meaning-bearing units relevant to the purpose of the study were condensed and then were subjected to further abstraction, and then named with codes. Further, codes were discussed with LTL to ensure the trustworthiness of the data and rigor. The codes were then compared with one another to identify differences and similarities. They were then given new names as appropriate. The codes that expressed similar patterns were clustered into subcategories and given new names. The researchers discussed their rational for making their specific decisions and their individual conclusions. This made it possible to name the subcategories that could then finally be reduced into categories. In this study, the authors pre-understanding was based only on GBP as a phenomenon, and this was not seen as affecting the results. Table 1 shows an example of the data analysis process.

**Table 1:** An example of the data analysis process

*Meaning unit*	*Condensed meaning unit*	*Code*	*Subcategory*	*Category*
‘… what I think is that at the specialist maternity care center they knew their thing or figured it out, while the conventional maternal health care was not so well prepared.’	Specialist maternity care center knew their thing	Expert at specialist maternity care center	The dedicated, knowledgeable, and skilled midwife	Importance of support
‘… when I asked if they have special guidelines for people who've gone through GBP surgery, they replied nothing they have made any note of. I felt this was bad because there should be some structure around those pregnancies …’	I felt it was bad because they had no special guidelines or structure	No guidelines or structure	Lack of support and knowledge	

An advisory statement was provided prior to the study by the Advisory Committee for Health Sciences Ethics at Lund University (VEN 79-17). Data were securely stored according to standard university data storage protocols, processed with confidentiality and anonymity was protected.

## RESULTS

The participant’s ages ranged from 23 to 36 years and all women had given birth during 2008–2017. The nine participants who had had more than one child had gone through GBP before the latest pregnancy. From the findings in this study, three main categories emerged which in turn could be unfolded into six subcategories (Table 2).

**Table 2:** Categories and subcategories

*Importance of support*	*The presence of the baby in the womb*	*Aggravating circumstances*
The dedicated, knowledgeable, and skilled midwife	Happiness in pregnancy	Fear of weight gain
Lack of support and knowledge	Concern for the baby’s health	Gastrointestinal complications

### Importance of support

The first main category ‘Importance of support’ included two subcategories: ‘The dedicated, knowledgeable, and skilled midwife’ and ‘Lack of support and knowledge’ describing the dualism of strengthening support from attending midwives during pregnancy and birth, but also how lack of support and knowledge from midwives could impact how pregnancy was experienced by a woman with prior GBP surgery.

*The dedicated, knowledgeable, and skilled midwife*

A dedicated, knowledgeable, and skilled midwife was described by the women as being responsive, supportive, and attentive. Strengthening support was experienced through her presence and interest. A skilled midwife, if previously inexperienced regarding GBP surgery, had read local guidelines directly after the first meeting and was aware of the implications of GBP surgery for pregnancy and could therefore support the woman in getting the appropriate care, thus being reassuring for the women:

*‘My midwife was very dedicated and followed [local] guidelines to the letter.’* (Anna)

The affirmative feeling of being seen and being heard was evident in the women’s narratives. All women appreciated the midwives’ involvement, especially when they took the time to listen. Having a midwife who was knowledgeable about GBP surgery in relation to pregnancy created security and trust in women and they described this as a prerequisite for good communication:

*‘It was great, she was fantastic all the time. She checked things up, she didn't know that much about GBP before, but next time I was there she was really up-to-date, it felt great.’* (Nadja)

The midwife was described as skilled when she knew that the oral glucose tolerance test offered in pregnancy is not carried out following a prior GBP surgery. Women experienced good support when the midwife’s professional skills were used to assess and refer to the specialist maternity healthcare when necessary or to assess if sick leave was appropriate and could then help to organize this. Being able to rely on the midwife was expressed as important:

*‘It has been really great! She is actually ... my midwife in X, where I attend, she is very familiar with this, with GBP. She knows what applies and that feels safe.’* (Kim)

The midwife’s skills and knowledge were crucial to the women’s experiences; in particular, the midwife at the specialist maternity healthcare center was experienced as the most knowledgeable and the women felt that the midwives had a broad internal network:

*‘... at the specialist maternity care center ... she was extremely skilled and extremely well informed … and the close collaboration with the doctor there who was also very skilled ...’* (Stina)

*Lack of support and knowledge*

Some women expressed that if the midwife in the conventional maternal healthcare system was not aware of the consequences of GBP surgery for the pregnant woman, the midwife’s knowledge was insufficient. Not having a midwife to lean on when complications arose was problematic. A midwife’s lack of knowledge created feelings in the woman of not being in control and not being involved in decisions. The experience of lack of support led to feelings of being disregarded by the midwife, which led to the experience that the conventional maternal healthcare system could not take care of the women or provide adequate care:

*‘… every time I had been there [at the antenatal clinic] I had to explain that I had gone through GBP surgery … they were quite lost because they lacked knowledge about the topic …’* (Lilly)

The women expressed disappointment when they felt they were not being taken seriously when they experienced severe pain. These women said that they had to contact their GBP surgeon when they did not receive satisfactory attention from midwives and doctors in connection with ileus. They also expressed a problem in their contacts with bariatric surgeons who had no knowledge of pregnancy:

*‘… when I asked if they have special guidelines for people who've gone through GBP surgery, they replied nothing they have made any note of. I felt this was bad because there should be some structure around those pregnancies…’* (Greta)

Almost half of the women felt that they themselves had to be updated regarding the care they needed when the midwife lacked knowledge:

*‘What I lacked was support and knowledge, really because it was only when I came to the specialist maternity care center ... then they had a bit more [to offer] than my usual antenatal clinic ...’* (Lisa)

### The presence of the baby in the womb

The second main category ‘The presence of the baby in the womb’ included two subcategories: ‘Happiness in pregnancy’ and ‘Concern for the baby's health’ describing the mother’s relationship with the baby in the womb, and how the health of the baby was of continual concern for the mother.

*Happiness in pregnancy*

One woman described how ‘time was ticking away’ and that the GBP surgery had been a first step in achieving pregnancy for many of the participants who expressed a profound joy in being able to conceive, especially when this occurred within a short time after the surgery. Even when experiencing complications, women were grateful and happy to become a parent, and women who became pregnant before the recommended time after surgery expressed contentment with being pregnant. Becoming pregnant spontaneously was perceived as giving them great happiness:

*‘… but it is so fortunate that I could get [pregnant] on my own after the surgery ... thanks to the surgery I have become pregnant. I couldn't before it.’* (Tanja)

*‘… happily, I got pregnant much faster after the surgery than before … I only got pregnant with the first child after trying to conceive for more than a year but after surgery then it took maybe 4–5 months or something like that…’* (Greta)

*Concern for the baby’s health*

Concerns about the baby’s health included worry about lack of nutrition and uncertainty about whether the baby had grown as expected. If the women could not eat, there was concern whether the baby would get the nutrition it needed. The women were aware of the examinations that were carried out in addition to the normal pregnancy examinations and interpreted this as an indication that the baby was at increased risk of being born small for gestational age. If the woman had a complication, there was concern that the baby would be affected and would be born premature. When the baby then was assessed as healthy and growing normally, the women expressed a feeling of relief:

*‘... because the slits [During the GBP surgery pockets and slits are formed between the gut segments] that held together had broken down, then I was in severe pain and could not eat so they [physicians] had to go in and operate, but the baby did well anyway.’* (Nadja)

Many of the women described how they experienced a lot of prejudice about their eating and there was a fear of being misinterpreted, a fear that others would think they did not eat enough food. The women spoke about insults and anxiety from the family who were afraid that the baby would be malnourished. This affected the women negatively:

*‘Because she was so tiny [in the womb] I was very worried that people, doctors, and midwives would get this impression that I did not eat, that is, it was the biggest fear.’* (Stina)

*‘People in my surroundings were very concerned since I was eating very little, they were concerned about the baby being fed what it needed…’* (Jessica)

### Aggravating circumstances

The third and last main category ‘Aggravating circumstances’ included the subcategories: ‘Fear of weight gain’ and ‘Gastrointestinal complications’ which explained circumstances that the women described as making pregnancy a challenging experience that became an emotional journey of fear and physical conditions including gastrointestinal complications.

All the women expressed how fear of regaining weight affected their everyday lives and that they feared returning to being the person they were before the surgery. They expressed a feeling of uneasiness in the beginning of pregnancy when weight gain could not be related to pregnancy. On the other hand, when the baby began to kick in the womb and the changed body image could be linked to the pregnancy, this concern decreased:

*‘I didn't want to go back being “Thick-Anna”. But when I started to feel the baby kick, I could relax and more easily accept the weight gain.’* (Anna)

The women stated that they needed support from their midwives in handling their fear of weight gain and said that midwives ought to be educated and have specific skills regarding pregnancy after prior GBP surgery and the special situation women can be in:

*‘I got really scared when I started to gain in weight when I was pregnant ... when you've lost a lot of kilos then you are afraid of becoming fat again as well ... it is good if they [midwives] become educated or know what to say in order to support women in my situation.’* (Inez)

*Gastrointestinal complications*

The women described how gastrointestinal complications such as ileus or dumping syndrome could cause a great deal of pain. Ileus must usually be addressed with surgery and undergoing an intervention during pregnancy was considered challenging. In cases where an emergency operation was not required, a sense of uncertainty that surgery could become necessary at any time ensued. For some women who did not undergo emergency surgery, labor was induced at term due to the pain. The pain and the uncertainty of the situation made the pregnancy difficult. The women stated that it was difficult to eat and that they were tired and felt debilitated. They needed to take analgesic tablets, and uncertainty about a possible emergency operation created great concern:

*‘... it can be surgery at any time on that part of the stomach, so it is trying and then being pregnant as well ...’* (Lilly)

Having more frequent occurrence than usual of ‘dumping’ during pregnancy resulted in discomfort including cold sweats, palpitations, stomach pain, nausea, and fatigue. Food cravings were experienced differently, and some women said that they ate more than normal and experienced more ‘dumping’. The feeling of satiety was experienced differently during pregnancy and contributed to more unrestrained eating:

*‘I experienced much dumpings during pregnancy just because I wanted to eat something then that I shouldn't eat. So, after I had eaten, I had to rest so it would pass.’* (Olga)

## DISCUSSION

In this interview study, women who became pregnant after prior GBP surgery described ambivalent feelings about their pregnant selves. They said that weight gain during pregnancy triggered memories of being obese, which is in line with Lier et al.^14^ who reported that fear of regaining weight is based on fear of returning to be the person the woman was before the GBP surgery. It takes several years to process and accept the new body after a weight loss following GBP surgery^14,15^. The weight gain was perceived as losing control of the body and one’s self-identity. It has been shown, in a recent prospective cohort study including 127 pregnant women, that gestational weight gain after bariatric surgery can have drawbacks. The study reported insufficient weight gain in almost 24% of the included women and 56% had excessive weight gain, which illustrates that there can be a struggle in finding a balance in food intake^16^. On the other hand, the women experienced that a spontaneous pregnancy was an assurance that their body could function normally. Intellectually, they understood the reason for not becoming pregnant too quickly after surgery but being able to conceive brought forth strong feelings of joy and happiness, particularly if they became pregnant shortly after the surgery. This was true even though they had been advised to wait for at least one year^17^. A cross-sectional survey had highlighted the need for more knowledge regarding women’s wishes concerning pregnancy after GBP surgery^18^. However, among the women in the present study, an understanding of GBP’s impact on the success of becoming pregnant was expressed. It can be speculated that in the setting the study was conducted in, young women have met midwives at youth clinics from early adulthood and learned that they could feel free to discuss future reproductive wishes whenever they wanted to. Women of reproductive age with morbid or severe obesity or with a history of GBP surgery may benefit from pre-conception counseling tailored to their reproductive wishes and should be offered this possibility in order to optimize both the experience of future pregnancy and obstetric outcome^19,20^.

The participants expressed a dualistic experience about consulting a midwife during pregnancy. The midwife’s attitude towards women who had undergone a pre-pregnancy GBP was crucial to the perceived support. The women felt strengthening support from a sensitive and attendant midwife. A committed midwife with an individualized, non-judgmental approach to care allows creation of a relationship between the midwife and the pregnant woman^21^. Lunda et al.^22^ reaffirmed that kindheartedness and sincerity from a midwife facilitate the development of a trusting relationship with the woman. It is evident that building up a midwife–woman relationship with continuous support may improve maternal and infant outcomes also for women who have undergone prior GBP surgery and their infants^23^.

A skilled and knowledgeable midwife knows that the body is an important component of self-identity in women negatively affected by changes to their body during pregnancy^24^. In addition, midwives, whose hands are their tools, must acknowledge that touching a pregnant woman’s body with their hands can cause discomfort for the woman. The midwife must obtain consent from the woman before providing tangible care^15^. Talking about weight gain early in pregnancy and examining how women with a history of GBP surgery relate to weight gain during pregnancy is of great importance, as weight gain in these women can arouse concern and fear^25,26^.

It was also important for the participants that the midwife had up-to-date knowledge about GBP surgery issues relating to pregnancy so that the well-being of these mothers could be optimized. Not only does the weight gain impact the woman’s experience of the pregnancy, but she also has specific concerns about the expected baby, as a fetus conceived after a prior GBP surgery may suffer from growth restriction, due to malabsorption, which is a common complication^9^. The midwife must be responsive when caring for women who have undergone prior GBP surgery. When feeling misunderstood without influence on decisions regarding their care in pregnancy, pregnant women felt powerless. Their vulnerability increased the sense of not being seen and taken care of^27^. If the midwife lacked knowledge, the women themselves felt responsible for being updated regarding guidelines and possible physical complications. This fact gave rise to feelings of insecurity and was experienced as lack of support. In the present study, both women who had been cared for by midwives at conventional antenatal clinics and those who attended specialist antenatal clinics were interviewed. York et al.^28^ reported that women with special needs experienced negative aspects as lack of knowledge and support by the midwife at the antenatal clinics. Midwives in conventional antenatal clinics may not have sufficient knowledge about GBP surgery in relation to pregnancy. Women-centered care, designed for pregnant women who have undergone GBP surgery and provision of general guidelines regarding care post GBP during pregnancy, birth and postpartum, will make this period less stressful for all.

### Strengths and limitations

This present study may be the first to address women’s experiences regarding pregnancy after GBP surgery from a qualitative perspective. Recruiting informants via social media is a growing trend^29^. Most informants were recruited via Facebook and these informants may not be fully representative of all pregnant women who have undergone GBP surgery. This limits transferability of the findings. On the other hand, social media are widely used by the age category that the included women belong to, and in addition recruitment via social media allows inclusion from a wider geographical area with diverse social population, thus enhancing transferability. It could be discussed whether time from surgery to birth to the time of the interview had an impact on the informants’ experience. The women in this study had had a GBP surgery not more than 8 years before the interview. However, it is well-known that women’s perception of pregnancy and birth are vivid and deeply felt^30^. Trustworthiness was strengthened by using direct quotations. In qualitative research, generalization of findings may never be fully justified as they, per se, will always be embedded within a specific context. Nevertheless, readers may decide for themselves if findings make sense in other contexts^31^. The women were interviewed by the two authors (AS and AM) who at the time of the interviews were registered nurses and midwifery students; however, reliability was strengthened by the senior researcher (LTL) who has extensive experience of content analysis. All researchers read the interviews, analyzed, discussed, and reflected upon the findings. None of the researchers had specific prior experience of antenatal care of women who had undergone GBP but have cared for women with this experience during labor and birth.

## CONCLUSIONS

Despite feeling ambivalent towards the weight gain and the possible physical complications during pregnancy, women who had undergone pre-pregnancy GBP surgery experienced great joy in being able to conceive and become pregnant spontaneously. A dedicated, knowledgeable, and skilled midwife strengthened the self-identity as a pregnant woman.

More knowledge is needed in how GBP surgery affects the woman emotionally when becoming pregnant and how antenatal care optimally can be provided to meet the increasing number of women becoming pregnant after GBP surgery. Employers should ensure highly competent midwives to care for pregnant women who have undergone GBP surgery. To increase the knowledge on women’s experience of being pregnant as well as during birth and postnatally after GBP surgery, more research with a qualitative design is needed, as there currently is a large research gap on the topic.

## Data Availability

The data supporting this research are available from the authors on reasonable request.

## References

[1] Mulla CM, Middelbeek RJW, Patti ME (2018). Mechanisms of weight loss and improved metabolism following bariatric surgery. Ann N Y Acad Sci.

[2] Akhter Z, Rankin J, Ceulemans D (2019). Pregnancy after bariatric surgery and adverse perinatal outcomes: A systematic review and meta-analysis. PLoS Med.

[3] Jastreboff AM, Kotz CM, Kahan S, Kelly AS, Heymsfield SB (2019). Obesity as a Disease: The Obesity Society 2018 Position Statement. Obesity (Silver Spring).

[4] Stubert J, Reister F, Hartmann S, Janni W (2018). The Risks Associated With Obesity in Pregnancy. Dtsch Arztebl Int.

[5] Carlson NS, Leslie SL, Dunn A (2018). Antepartum Care of Women Who Are Obese During Pregnancy: Systematic Review of the Current Evidence. J Midwifery Womens Health.

[6] Johansson K, Cnattingius S, Näslund I (2015). Outcomes of pregnancy after bariatric surgery. N Engl J Med.

[7] Stenberg E, Chen R, Hildén K, Fall K (2020). Pregnancy As a Risk Factor for Small Bowel Obstruction After Laparoscopic Gastric Bypass Surgery. Ann Surg.

[8] Jensen JF, Petersen MH, Larsen TB, Jørgensen DG, Grønbaek HN, Midtgaard J (2014). Young adult women's experiences of body image after bariatric surgery: a descriptive phenomenological study. J Adv Nurs.

[9] Kwong W, Tomlinson G, Feig DS (2018). Maternal and neonatal outcomes after bariatric surgery; a systematic review and meta-analysis: do the benefits outweigh the risks?. Am J Obstet Gynecol.

[10] Elizabeth McGuire E (2018). Nutritional consequences of bariatric surgery for pregnancy and breastfeeding. Breastfeed Rev.

[11] (2016). WHO recommendations on antenatal care for a positive pregnancy experience.

[12] Graneheim UH, Lundman B (2004). Qualitative content analysis in nursing research: concepts, procedures and measures to achieve trustworthiness. Nurse Educ Today.

[13] Newington L, Metcalfe A (2014). Factors influencing recruitment to research: qualitative study of the experiences and perceptions of research teams. BMC Med Res Methodol.

[14] Lier HØ, Aastrom S, Rørtveit K (2016). Patients' daily life experiences five years after gastric bypass surgery--a qualitative study. J Clin Nurs.

[15] Nyman VM, Prebensen AK, Flensner GE (2010). Obese women's experiences of encounters with midwives and physicians during pregnancy and childbirth. Midwifery.

[16] Ceulemans D, De Mulder P, Lebbe B (2021). Gestational weight gain and postpartum weight retention after bariatric surgery: data from a prospective cohort study. Surg Obes Relat Dis.

[17] Pg Baharuddin DM, Payus AO, Abdel Malek Fahmy EH (2021). Bariatric surgery and its impact on fertility, pregnancy and its outcome: A narrative review. Ann Med Surg (Lond).

[18] Mengesha BM, Carter JT, Dehlendorf CE, Rodriguez AJ, Steinauer JE (2018). Perioperative pregnancy interval, contraceptive counseling experiences, and contraceptive use in women undergoing bariatric surgery. Am J Obstet Gynecol.

[19] Falcone V, Stopp T, Feichtinger M (2018). Pregnancy after bariatric surgery: a narrative literature review and discussion of impact on pregnancy management and outcome. BMC Pregnancy Childbirth.

[20] Farahi N, Zolotor A (2013). Recommendations for preconception counseling and care. Am Fam Physician.

[21] Dencker A, Premberg Å, Olander EK (2016). Adopting a healthy lifestyle when pregnant and obese - an interview study three years after childbirth. BMC Pregnancy Childbirth.

[22] Lunda P, Minnie CS, Benadé P (2018). Women's experiences of continuous support during childbirth: a meta-synthesis. BMC Pregnancy Childbirth.

[23] Bohren MA, Hofmeyr GJ, Sakala C, Fukuzawa RK, Cuthbert A (2017). Continuous support for women during childbirth. Cochrane Database Syst Rev.

[24] Brown A, Rance J, Warren L (2015). Body image concerns during pregnancy are associated with a shorter breast feeding duration. Midwifery.

[25] Christenson A, Johansson E, Reynisdottir S, Torgerson J, Hemmingsson E (2018). Shame and avoidance as barriers in midwives' communication about body weight with pregnant women: A qualitative interview study. Midwifery.

[26] Epiphaniou E, Ogden J (2010). Successful weight loss maintenance and a shift in identity: from restriction to a new liberated self. J Health Psychol.

[27] Ryding EL, Wijma K, Wijma B (2000). Emergency cesarean section: 25 women's experiences. J Reprod Infant Psychol.

[28] York S, Briscoe L, Walkinshaw S, Lavender T (2005). Why women choose to have a repeat caesarean section. Br J Midwifery.

[29] Brandão C, Silva R, dos Santos JV (2019). Online recruitment in Portugal: Theories and candidate profiles. J Bus Res.

[30] Simkin P (1991). Just another day in a woman's life? Women's long-term perceptions of their first birth experience. Part I. Birth.

[31] Polit DF, Beck CT (2010). Generalization in quantitative and qualitative research: myths and strategies. Int J Nurs Stud.

